# Multilevel analysis of dendroclimatic series with the R-package BIOdry

**DOI:** 10.1371/journal.pone.0196923

**Published:** 2018-05-17

**Authors:** Wilson Lara, Stella Bogino, Felipe Bravo

**Affiliations:** 1 Sustainable Forest Management Research Institute, UVA-INIA, Avda. Madrid, s/n, 34071, Palencia, Spain; 2 Department of Geography and Urban Studies, College of Liberal Arts, Temple University, 316 Gladfelter Hall, 115 W. Polett Walk, Philadelphia, United States of America; 3 Departamento de Ciencias Agropecuarias, Universidad Nacional de San Luis, Avenida 25 de Mayo 384, 5730, Villa Mercedes, San Luis, Argentina; Pacific Northwest National Laboratory, UNITED STATES

## Abstract

The R-package BIOdry allows to model and compare fluctuations of Tree-ring Width (TRW) and climate, or dendroclimatic fluctuations, while accounting for source variability. The package eases multilevel modeling and multivariate comparison in dendroclimatic analysis using the nlme and ecodist packages, respectively. For implementing such libraries, the in-package algorithms transform the dendroclimatic fluctuations into Multilevel Dendroclimatic Data Series and maintain categorical variables and time units in the outputs. The dendroclimatic modeling is developed with two functions: modelFrame and muleMan. The first function binds core-level cumulative TRWs to the processed data sets and subtracts trends in TRWs by fitting multilevel log-linear growth formulas or multilevel linear formulas. modelFrame can also model within-group fluctuations in dendroclimatic variables other than tree-radial increments such as aridity indices or allometric components of tree growth: e.g. diameters at breast height over bark, tree basal areas, total tree biomass, among other. The second function compares fluctuations in modelFrame objects that share outermost categorical variable and annual records. Here, we use BIOdry to model dendroclimatic relationships in northern and east-central Spain to illustrate future users in the implementation of the package for modeling ecological relationships in space and time.

## 1 Introduction

Dendrochronological studies date and analyze tree-rings from woody plants to understand past and current environmental processes. Dendroclimatology uses tree rings to study past climate conditions. It is one of the most prominent dendrochronological application to study global climate change [1]. Most standard approaches to model Tree-ring Width (TRW) and climate fluctuations implement time-series decomposition at specific levels of variance (single-level modeling) [2, 3]. The TRW data extraction usually requires sampling with specialized hardware [4] and/or specific statistical software [2, 3, 5, 6]. These procedures have been efficiently implemented not only for climate change studies [1] but also in paleoclimatic reconstructions [1, 7–9], the assessment of forest response to water availability [10, 11], among others [12–15]. Even though these methods are widespread applied, they have not always considered the sampling design and the different levels of variation in tree-ring series. Sampling dendroclimatic variables in forest ecosystems results in hierarchical sources of variability from ecological factors. For instance, variability in core replicates (number of samples per each analyzed tree) of TRW from forest communities would have at least three hierarchical levels: tree-radial morphology [16], individual-tree genetics or phenotype [17], and stand quality [15]. Additional ecological factors such as site elevation [18], fire intensity [14], tree decay [19], or water regimes [10, 11] can further complicate hierarchical variance in TRW. Meteorological records, which are used to model climatic variables in dendroclimatic analyses, also contain temporal and spatial variability [20, 21].

Developing software for dendroclimatic analysis in forest ecosystems can help to address these sources variability. This new software should account for the effects of both ecological-factors and sampling schemes, and should integrate tree-growth/yield modeling into dendroclimatic analysis [22–24] based on multilevel modeling and multivariate comparison.

One example of multilevel modeling methods is mixed-effects regression [25–28], which can be implemented to detrend dendroclimatic data by considering random effects from ecological factors. Another example is dissimilarity analysis [29–32], which can be used to compare and organize dendroclimatic fluctuations into common ecological-factor levels. However, the implementation of these methods using statistical environments requires dendroclimatic inputs stored in special formats [33] such as Multilevel Dendroclimatic Data Series (MDDS), or sequences of observations ordered according to spatial/temporal hierarchies which are the result of sampling schemes, with sample variability confined to ecological factors.

The dendroclimatic modeling in forest ecosystems should also integrate information about tree allometry, growth, and yield. For example, allometric scaling can help transform TRW data into other serial components of tree growth [34–38]. Similarly, organic growth theory has provided simplified equations [39–41] that can be used to subtract ontogenetic trends from TRW data. Such equations are log-linear expressions of tree growth that are easily fitted to the multilevel data applying mixed-effects-modeling procedures: e.g. lme methods in R [42].

In addition, dendroclimatic modeling can also involve time-series transformations at specific levels in the ecological factors. Such modeling is important for evaluating allometric parameters for TRW [36–38], computing water-availability indexes from climatic MDDS [20, 43–45], developing statistical process control for TRW series [2, 46–48], or for time-series smoothing and decomposition [49, 50]. Procedures to evaluate this kind of routines should be efficient and preserve MDDS structures and be adaptable to new methods in dendroclimatic modeling. Consequently, efficient implementation of all these procedures requires of programming higher-order functions which can recursively evaluate diverse routines, control multilevel detrending, and compare fluctuations in MDDS.

In this work, we present the R-package BIOdry, a statistical package that processes MDDS using higher-order functions to integrate processes for input derivation, multilevel analysis, and multivariate comparison of dendroclimatic fluctuations. Here, we explain the functionality of the package by modeling relationships between TRW fluctuations and drought using MDDS from pine forests in the Iberian Peninsula. We also test the hypothesis that accounting for ecological factors improves statistical analysis for dendroclimatic modeling compared to other conventional alternatives, such as linear regression. To do so, we compare parameters from implementing two procedures: conventional single-level modeling and the multilevel procedures in the package. The examples of dendroclimatic modeling developed here are also intended as a guideline for future BIOdry users interested in implementing the package for modeling other ecological relationships in time and space.

## 2 Package installation and requerimients

The primary focus of this paper is on BIOdry 0.5 (R versions ≥ 3.4.2). This and further versions of the package can be easily installed in any R session using the install.packages command. Once installed, the package is loaded in the R environment with the library command. The in-package routines are programmed with generic functions in base, stats and rutils packages. Functions for developing the multilevel modeling and the multivariate comparison depend on two R packages in CRAN: nlme [42], and ecodist [30]. These are automatically loaded in R after the package has been requested. A further dependence, the nlme package, is used to extract fluctuations in MDDS, or Multilevel Dendroclimatic Data Series, with linear mixed effects models (detrending); while ecodist is used to compute multivariate correlations (Mantel correlograms) between two compared MDDS.

### 2.1 Data inputs

BIOdry processes three kind of data sets: TRW (mm), monthly cumulative precipitations (mm), and monthly average temperatures (°C). These can have wide or long formats. In the wide data format, the rows are ordered and labeled from earliest to latest year and the columns contain the dendroclimatic variables referred above. Names of the columns must be dot-separated codes representing the hierarchy of ecological factors, where higher ecological levels are defined first and lower levels after. For instance (truncations are indicated with ellipses):

> data(Pchron)

> tail(Pchron, 3)

     P16106.17.a   P16106.17.b… P44005.7.b

2003       0.926          0.882…      0.621

2004       1.205          0.638…      0.368

2005       0.964          0.295…      0.038

Pchron is a TRW (1861-2005) for *Pinus pinaster* Ait. forests (*P*. *pinaster*) in northern and east-central Spain [51]. These two regions have contrasting climate regimes with forests in central areas of the country being more affected by drought than forests in northern regions. To account for source variability, two trees were selected per site, and two cores were extracted from each tree. Consequently, the column names have within-plot levels representing variabilities in the plot qualities (alphanumerics), tree genetics/phenotypes (numbers), and tree-radial morphology (lowercase letters).

Meteorological records in wide formats have the rows labeled with years and the columns labeled with dot-separated codes of nearby plots and monthly abbreviations. For instance:

> data(Prec)

> data(Temp)

> names(Prec) # or names(Temp)

[1] “P16106.Apr” “P16106.Aug” “P16106.Dec” “P16106.Feb”…

The Prec sample data set, included in BIOdry, consists of cumulative precipitations, while Temp contains corresponding monthly temperatures. These were provided by the Spanish Meteorological Agency (AEMET).

On the other hand, long data formats should have the chronologies and time units in the initial columns, followed by ecological factors. In the factor hierarchy, columns of lower time units or classification factor levels are defined first, and columns of higher levels are specified later. For instance,

> data(Prings05)

> head(Prings05, 3)

       x year sample tree    plot

1 0.698 1897      a    17 P16106

2 0.878 1898      a    17 P16106

3 0.842 1899      a    17 P16106

where Prings05 is a long format of data stored in Pcrhon.

An alternative vector of tree radii can also be specified although this is not mandatory. In this case, the year of measurement of the radii should also be recorded. Until recently, core samples often lacked the initial rings of the pits producing truncated growths [22]; so these reference radii can be used to scale the cumulative growth. This vector should be named with dot separated codes and contain codes in the tree factor of the TRW.

> data(Pradii03)

> Pradii03

P16106.17 P16106.18 P44005.56 P44005.7

 122.2343 139.7485 158.5184 114.9109

## 3 Package functionality

**Fig 1:**
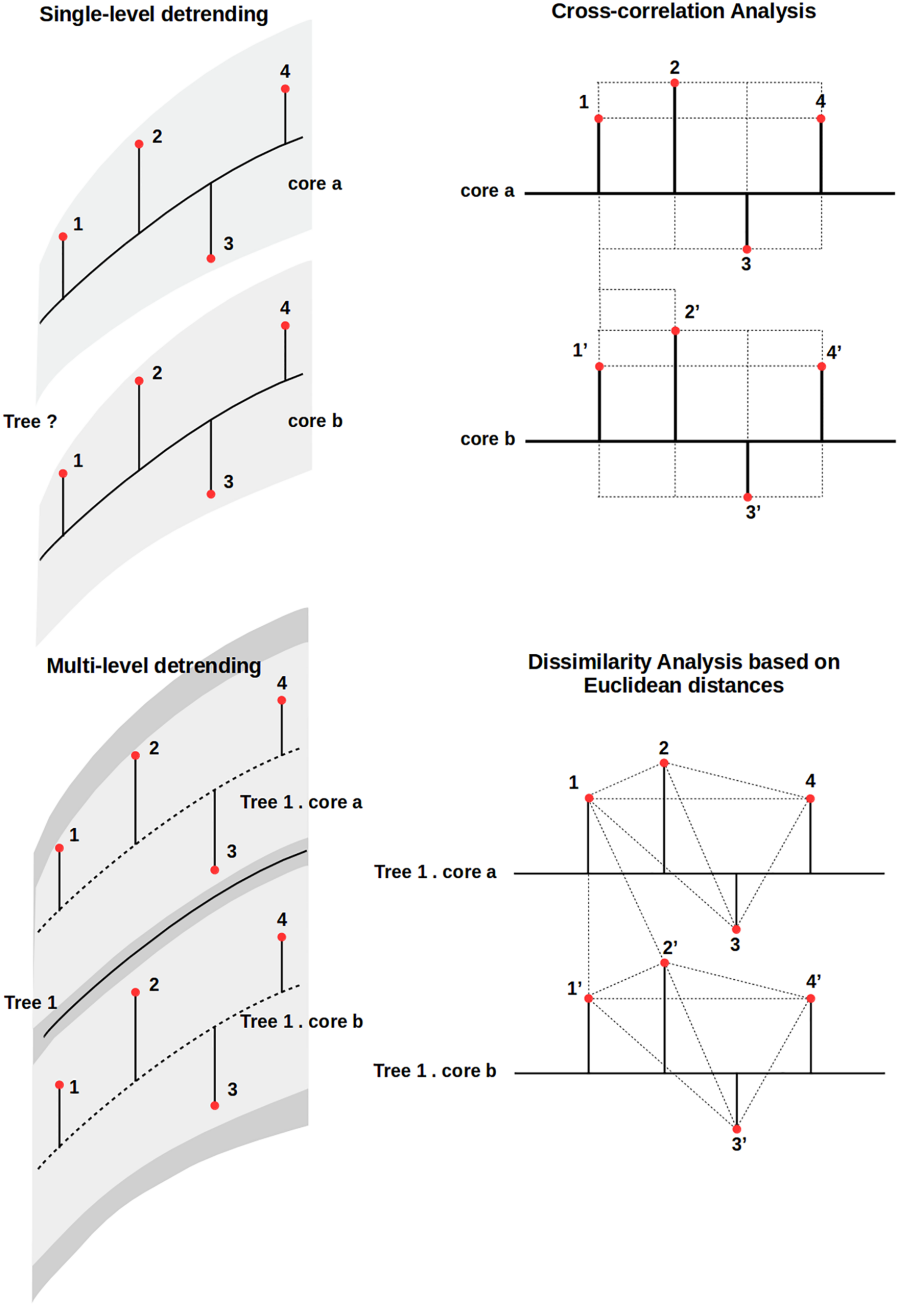
Comparison of procedures to model dendroclimatic fluctuations. Standard approaches (upper panels) usually account for sample variability (gray areas). These implement cross-correlation analyses to compare the detrended fluctuations. On the other hand, the BIOdry package (lower panels) consider hierarchical structures from sample design (gray layers) to account for source variability: e.g. tree morphology. The package implements dissimilarity analysis to compare the detrended fluctuations.

Dendroclimatic modeling has evolved from paleoclimatic sciences where the ecological variability is seen as noise [9]. Consequently, statistical procedures in dendroclimatic research traditionally consider ecological variability as irrelevant. These standard methods model signs by independently detrending TRW/climatic fluctuations (Fig 1, upper panels) and comparing the extracted signs with cross-correlation functions.

Procedures in the BIOdry package can complement these approaches by modeling and comparing dencroclimatic fluctuations while accounting for ecological variability (Fig 1, lower panes). To do so, the package implements methods to derive, detrend and correlate multilevel dendroclimatic fluctuations while accounting for factors defined in MDDS (Appendix A). These are programmed using a variety of in-package routines (Tables 1 and 2) which could be implemented independently if users are interested in running specific calculations or better understanding the sequence of steps for dendroclimatic modeling. However, implementation of these can be burdensome. Consequently, the routines can also be recursively evaluated on MDDS using two higher-order functions: modelFrame and muleMan. Former modelFrame can either derive allometric components of tree-growth fluctuation from TRW data or compute aridity indexes from meteorologic records depending on whether defaults in the function are maintained or modified.

**Table 1:** Routines (rt) and detrending formula (df) used to model tree-growth fluctuations from TRW with modelFrame. These are specified in the form of lists with the fn and lv arguments (see second argument in Table 3). Arguments of these one-level functions are specified in modelFrame in either a MoreArgs list argument or directly, depending on whether they are vectors (e.g. mp) or constants, respectively.

Type	Name	Details	Arg.	Arg. definition
**rt**	rtimes	Unique observations in time-units data with replicates (time-series replicates) are excluded to avoid biases during subsequent multilevel detrending [22].	only.dup = TRUE	logical. Extract only relative times that are duplicated. If TRUE then unique observations are replaced with NA. If all computed times are unique then this argument is ignored. This function binds to the processed data set a new column time.
**rt**	scacum	Cumulative sums of time-series replicates (e.g. radial increments) are scaled on reference constants (e.g. individual tree diameters).	sc.c = NA	numeric. Scaling constant, or vector if the processed time-series replicates have several levels, to scale the computed cumulative values. If NA then the computed cumulative sums are not scaled.
			rf.t = NA	numeric. Constant or vector of reference time(s) in range(s) of the vector names to scale the cumulative values. If NA then maximum value in the range is used.
**rt**	amod	Simple allometric model: *y* = *a* ⋅ *x*^*b*^ is recursively evaluated to derive allometric components of organisms from longitudinal variables (e.g. Cumulative radial increments).	mp = c(1, 1)	numeric vector with allometric parameters: *a*, *b*. Default maintains the original radii, c(2,1) produces diameters, and c(0.25 * pi,2) computes basal areas. Further parameters of tree volume or biomass equations can also be implemented. This argument can have more than two parameters: c(a1,b1,a2,b2,…,an,bn), with n being the number of times that the allometric model is recursively implemented.
			fun = y a*(x^b)	formula. Allometric model.
**df**	tdform	log-linear time-decline formula with random effects structure: ‘log(x) ˜ log(csx) + f(time) | group’; where x is the relative organic growth; csx is the cumulative organic growth; f(time) is a function of time.	on.time = TRUE	logical. If TRUE then t = ‘time’ (see rtimes function in this Table). If false then t = ‘year’
			log.t = FALSE	logical. If TRUE then f(time) = log(time) or f(time) = log(year) depending on the on.time argument.
			lev.rm = NULL	NULL or character vector. name(s) of the factor(s) to be removed from the group term.

**Table 2:** One-level functions (rt) and detrending formula (df) used to model AAI fluctuations from monthly average temperatures and monthly cumulative precipitations. Implementation of these is similar to what was explained for tree growth modeling in Table 1.

Type	Name	Details	Arg.	Arg. definition
**rt**	moveYr	Monthly records in time-series replicates (usually of climate) are labeled for the years can begin in a month other than January.	ini.mnt = ‘Oct’	character, or numeric from 1 to 12. Initial month of the seasonal year. If character then the months are built-in constants in R-package base. Default ‘Oct’ makes the years begin in October, for example.
**rt**	wlai	Annual aridity indexes from Walter-Lieth diagrams are computed from monthly precipitation sums and monthly average temperatures.	sqt = TRUE	logical. Print the square root of the aridity index. If TRUE then computed aridity index is normalized with a square root transformation.
**df**	lmeform	LME formula with random effects structure: ‘resp ˜ cov | group’; where resp is the response; cov is the primary covariate; and group is the random-effects structure.	resp = NULL	NULL or character. Column name of the response. If NULL then the name of the first numeric column in the MDDS is used.
			covar = NULL	NULL or character. Column name(s) of the covariate(s). If NULL then the name of the first time-units column in the MDDS is used.
			lev.rm = NULL	NULL or character vector. Name(s) of the factor(s) in the MDDS to be removed from the group term.

### 3.1 Modeling features

#### 3.1.1 Function defaults

By default, the modelFrame function synchronizes, cumulates, and detrends TRW by evaluating three routines and a detrending formula (Table 3). The routines are defined in the fn argument and vectorized over factors in the lv argument. The synchronization circumvents biases causing artificial increases or decreases in the modeled tree growth: slow-grower survivorship, big-tree selection, among other [22] while enhancing convergence of parameters during the detrending procedure [42]. The cumulative TRWs are used to model ontogenetic growth fitting detrending formulas [41]. These are specified with the form argument in modelFrame. The defaults are maintained by only specifying the processed MDDS. For instance,

**Table 3:** Formulation order of parameters in modelFrame. This function is used to model fluctuations of Tree-ring Widths (TRWs) (cm) and Annual Aridity Indexes (AAIs) (dimensionless).

Order	Arguments and defaults	Description
**1**	rd	Individual data.frame of TRW or twofold list of data frames with monthly cumulative precipitations and monthly average temperatures.
**2**	fn = list(‘rtimes’,‘scacum’,‘amod’)	list. character names of functions for one-level modeling (one-level functions) that are recursively implemented. See arguments in Tables 1 and 2.
**3**	lv = list(‘tree,‘sample’,‘sample’)	list. Either column names or numeric positions in the column factors in the MDDS that are used to evaluate the one-level functions.
**4**	Arguments and defaults in one-level functions	See one-level functions (**ofn**) and respective arguments in Tables 1 and 2.
**5**	form = ‘tdForm’	Detrending formulas (Tables 1 and 2)
**6**	Arguments in lme function	

> trwf <- modelFrame(Pchron)

On the other hand, muleMan compares TRW and aridity-Index fluctuations while maintaining hierarchical structures in the MDDS, see Section 7.1.

#### 3.1.2 Detrending formulas

Currently, the ‘tdForm’ or ‘lmeForm’ methods can be implemented automatically with the form argument to evaluate formulas with the same names. The ‘tdForm’ method is a linear generalization from growth theory (Appendix A.1) used here to subtract ontogenetic growth from TRW. This method considers either time units or classification factors in MDDS as random effects, see details about formulation of ‘tdForm’ method in Section A.1.2. The ‘lmeForm’ method implements a more flexible linear formula, which is implemented here to detrend normalized aridity indexes. See also structure of ‘lmeForm’ method in Section A.1.3.

#### 3.1.3 Correlation functions and variance structures

The package can implement procedures for serial normalization in the nlme package. Heteroscedasticity and serial autocorrelation of the detrended MDDS can be modeled with arguments in lme methods that are specified in modelFrame: e.g. weights and correlation arguments in the nlme package (Appendices A.3 and A.4). Likewise, autocorrelation in detrended MDDS can be assessed with empirical autocorrelation functions.

#### 3.1.4 Multivariate comparison

Mantel correlograms between two MDDS with a common classification factor are established by comparing distances in one of the MDDS with sets of binary model matrices that specify membership in the other MDDS classes (Appendix A.5). The multivariate comparison is implemented here to compare detrended aridity indexes with detrended TRW.

## 4 Tree-growth modeling and diagnostics

Parameters of tree growth can be enhanced by changing arguments of the routines in the modelFrame function or updating modelFrame objects with the new arguments. The new model updating can help for properly scaling truncated fluctuation [22] and correctly defining shape of tree ontogeny [41]. TRW lacking initial rings in the pits can be scaled around the vectors of reference radii specifying the tree radii and corresponding measurement year in sc.c and rf.t arguments, respectively. Likewise, the detrending formula can be modified from Chapman-Richards to Levakovic forms, changing default log.t = FALSE to log.t = TRUE respectively. The forms are simplified expressions of most growth equations used in forest sciences to model tree growth [41]. Here, we update the trwf object modifying these parameters with the update call:

> trwf <- update(trwf,

                    sc.c = Pradii03,

                    rf.t = 2003,

                    log.t = TRUE)

The trwf object contains a threefold list of model parameters in model, TRW fluctuations in fluc, and the model call in call. Parameters in trwf can be inspected calling the model element with currency or bracket operators and using plenty diagnostic functions in the nlme package [42]. Alternatively, we have developed five in-package S3 methods to directly inspect parameters in modelFrame objects with lme functions: summary, plot, getData, anova, and Empiric Autocorrelation Function (ACF). These are implemented here to inspect significances of fixed-effects parameters, compare models with similar fixed-effects structures, and detect serial patterns in normalized fluctuations.

### 4.1 Model diagnostics

The summary function provides information about the estimation method (default method = REML), information-based criteria (Akaike Information Criterion (AIC), Bayesian Information Criterion (BIC), and Log-Likelihood (logLik)), fixed-effects formula (default form = ‘tdForm’), random-effects standard deviations, and conditional t-tests, as shown in following summary output:

> summary(trwf)

Linear mixed-effects model fit by REML

 Data: fixed

         AIC       BIC     logLik

   1425.193 1488.797 -699.5963

Random effects:

Formula: ˜log(csx) + log(time) | plot

Structure: Diagonal

         (Intercept) log(csx) log(time)

StdDev: 0.9541355 0.00115044 0.226334

Formula: ˜log(csx) + log(time) | tree %in% plot

Structure: Diagonal

         (Intercept) log(csx) log(time)

StdDev: 0.872989 2.42083e-07 0.2220615

Formula: ˜log(csx) + log(time) | sample %in% tree %in% plot

Structure: Diagonal

         (Intercept)  log(csx) log(time) Residual

StdDev: 0.09011541 6.108796e-24 0.02288596 0.4784995

Fixed effects: log(x) ˜ log(csx) + log(time)

         Value Std.Error DF t-value p-value

(Intercept) -3.171448 1.4210373 978 -2.231784 0.0259

log(csx)  1.398107 0.3585760 978 3.899055 0.0001

log(time)  -0.953834 0.2296603 978 -4.153239 0.0000

Correlation:

      (Intr) lg(cs)

log(csx) -0.822

log(time) 0.423 -0.521

Standardized Within-Group Residuals:

     Min  Q1  Med  Q3  Max

-6.9065514 -0.5052164 0.1119620 0.6316315 3.5025194

Number of Observations: 988

Number of Groups:

          plot   tree %in% plot

              2                  4

sample %in% tree %in% plot

                              8

The random effects of the summary output define the limits of the hierarchical levels and are expressed in standard deviations. The fixed effects show conditional t-tests testing marginal significances of fixed effects. These suggest that all the fixed-effects terms of multilevel model in trwf are significant (p ≤ 0.026). We can infer that tree-growth parameters derived from the TRW conforms to the theoretical growth patterns implied by the Levakovic expression of tdForm. Diagnostic plots for assessing the quality of the fit are obtained using the method for the plot function.

**Fig 2:**
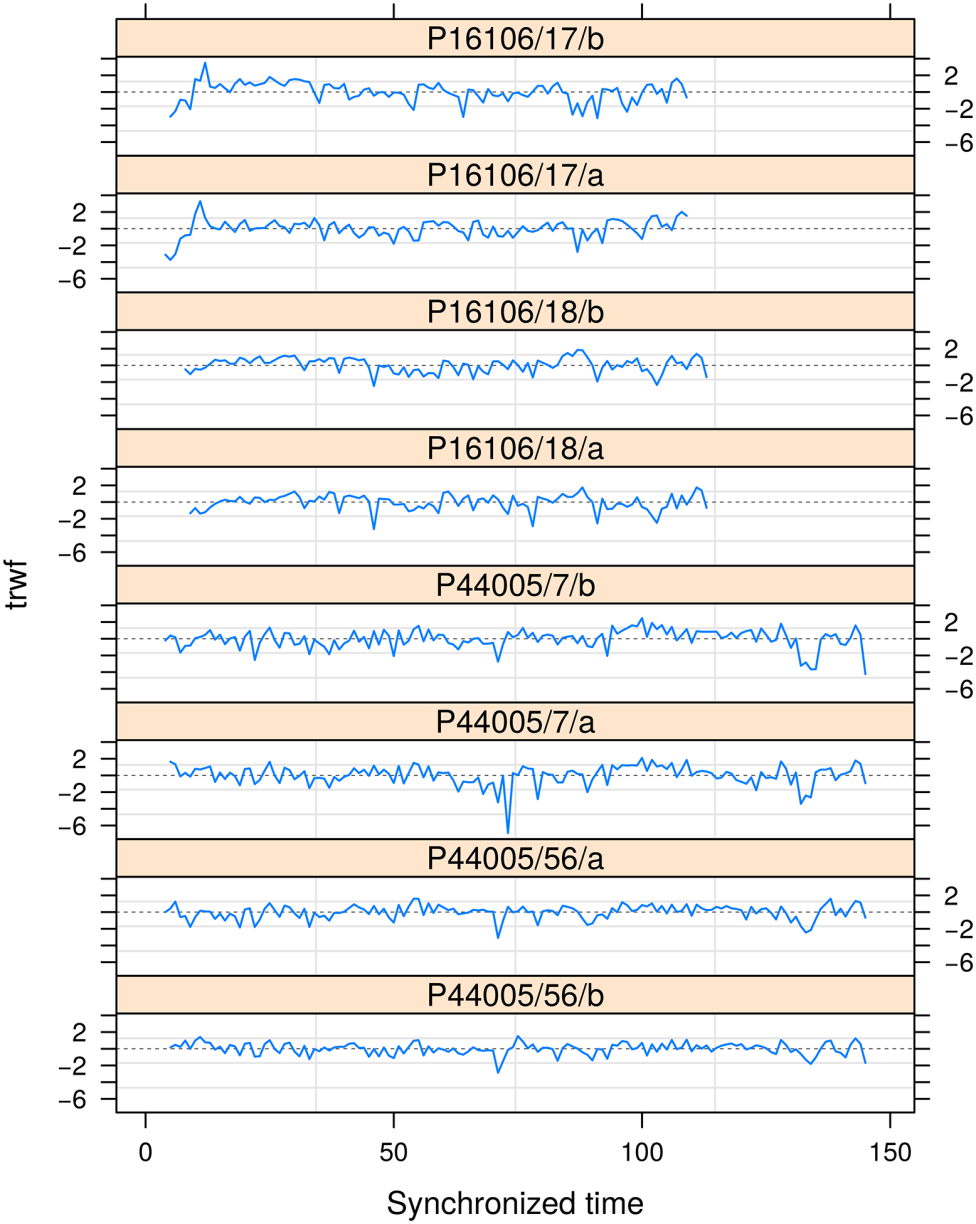
Fluctuations in the chronology of *P. pinaster*. Residual autocorrelations in the chronology has been accounted for with an auto-regressive structure for lags ≥ 2 (ARMA(p = 1, q = 1)) after normalizing the fitted residuals via Choleski factorization.

> plot(trwf) ## Fig (2)

Accounting for the within-plot variability affected patterns between the TRW fluctuations (Fig 2). Fluctuations from trees in similar plots were more comparable than these in different plots. This could be explained by the different biophysical conditions of the plots, for instance: genetic adaptations of the populations, local variations in nutrients and water availability, competition [52].

To analyze improvements in model fitting by accounting for within-core variability, we ignore the grouping structure of the tdForm formula and fit a single linear regression without random effects to the TRW data. The model is fitted with lm regression in default R-package stats. The cumulative TRW are recycled from the trwf object with getData function. Parameters fitted with the new lm model are compared with parameters of the multilevel model in trwf doing a log-Likelihood ratio test(logLik) with the anova function.

> trwfl <- lm(log(x) ˜ log(csx) + log(time),

         data = getData(trwf))

> anova(trwf,trwfl)

       Model df       AIC       BIC     logLik   Test     L.Ratio    p-value

trwf       1 13 1425.193 1488.797 -699.5963

trwfl      2  4 1525.847 1545.418 -758.9235 1 vs 2    118.6543     <.0001

This suggests that accounting for within-core variability significantly reduces AIC, BIC, and logLik (p ≤ 10^−4^). Consequently, we assert that multilevel model in trwf fits better than the tdForm model with only fixed effects.

### 4.2 Allometric components of tree grotwth

Allometric relationships in components of tree growth other than TRW: e.g. tree diameters, basal areas, or tree biomasses, have not been fully explored to understand non-linear relationships between tree growth and drought [53]. The modelFrame function can help to explore such relationships by recursively evaluating allometric parameters in mp and formatting metric units of the TRW in to argument. However, this recursive evaluation of allometric parameters must be done with care because excessive iteration of parameters could exponentially increase errors of the allometric models. Here, we specify two pairs of parameters in mp to compute fluctuations in Diameters at Breast Height over bark (DBH). The first pair of parameters c(2, 1) is used to compute diameters at breast height inside bark (dib) from the TRW. Metric units of the TRW are transformed from mm to cm before the dibs are computed. This is done by writing to = ‘cm’. The other two parameters c(2.87, 0.85) are used to predict the DBHs (cm) from the dibs. The four allometric parameters are formulated in a common vector inside a MoreArgs list. This avoids the modelFrame function to vectorize over the allometric constants along the within-core levels.

> tdf <- update(trwf,

                   to = ‘cm’,

      MoreArgs = list(mp = c(2, 1, 2.87, 0.85)))

> summary(tdf)

Linear mixed-effects model fit by REML

Data: fixed

        AIC       BIC     logLik

  1425.903 1489.507 -699.9515

#…

Fixed effects: log(x) ˜ log(csx) + log(time)

                    Value Std.Error DF    t-value p-value

(Intercept) -3.185970 1.3154323 978 -2.421995 0.0156

log(csx)      1.469242 0.4220967 978  3.480818 0.0005

log(time)    -0.954741 0.2298998 978 -4.152858 0.0000

#…

## 5 Random-effects structures

Hierarchical sources of variability from endogenous and exogenous disturbances in the stands are usually masked in the error terms of the TRW chronologies or the climatic fluctuations [54]. BIOdry implements methods to account for this variability using the modelFrame function. By default, this function constructs random effects with pdDiag constructor class in nlme [42], a primary covariate from the formula in form, and the factors in the MDDS. However, users of the package can specify other constructor classes, see p. 157 in [42], and different random-effects covariates while removing factors from the processed MDDS. These are specified in either the modelFrame or update functions with the random and lev.rm arguments. We update here the trwf object with new parameters to modify original random-effects structure in trwf by specifying the pdIdent constructor class and the ˜time covariate while removing the sample random effect.

> trwfr <- update(trwf,

            random = pdIdent(˜ time),

              lev.rm = ‘sample’)

> summary(trwfr)

Linear mixed-effects model fit by REML

 Data: fixed

         AIC        BIC    logLik

   1510.969 1540.325 -749.4844

Random effects:

 Formula: ˜time | plot

 Structure: Multiple of an Identity

       (Intercept)   time

StdDev: 8.272046e-07 8.272046e-07

 Formula: ˜time | tree %in% plot

 Structure: Multiple of an Identity

          (Intercept)         time Residual

StdDev: 0.001311275 0.001311275 0.512138

#…

## 6 Serial modeling

Multilevel analysis of dendroclimatic data involves spatial and temporal pseudoreplication: i.e., series in the hierarchical levels are correlated introducing dependencies among the modeled fluctuations [55]. BIOdry can print ACF plots to inspect such dependencies. The inspection process is controlled with two arguments: maxLag and alpha. The first argument controls the number of lags for which the autocorrelations are computed. The second argument specifies the significance level for two side critical bounds.

**Fig 3:**
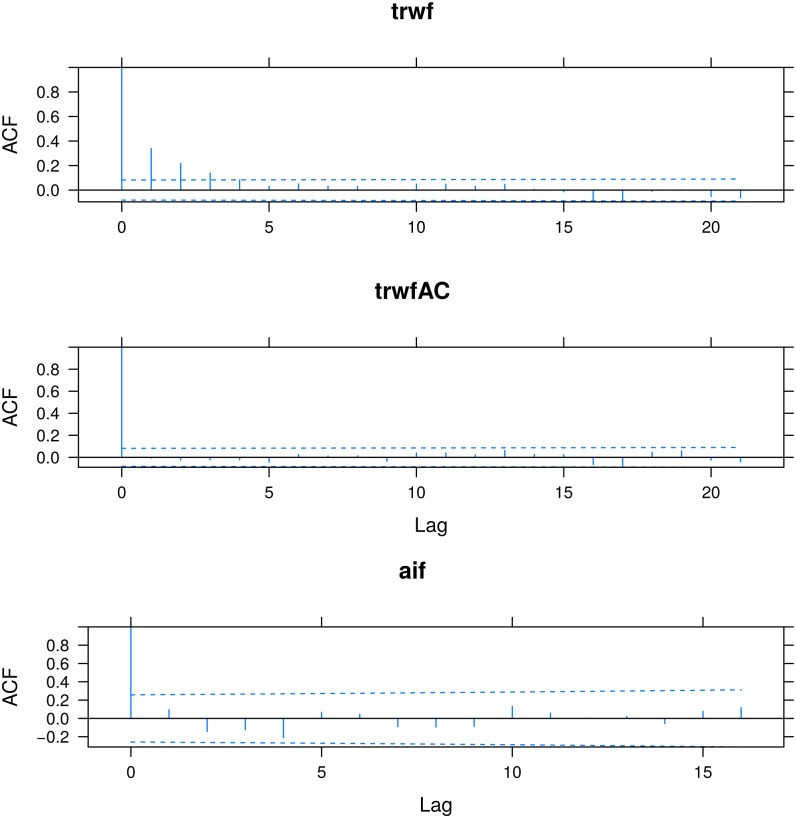
Empiric Autocorrelation Functions (ACFs) for the chronology of *P. pinaster* and the aridity-index fluctuations in three modelFrame objects. Labels indicate level-codes in the plot factor.

> plot(ACF(trwf, maxlag = 10),

      alpha = 0.01) ## Fig (3), upper panel

The plot shows that fluctuations in trwf are autocorrelated at three annual lags (Fig 3, upper panel). Part of this occurred because the core samples are replicated across the within-plot levels introducing dependencies among the residuals [55]. Another part of the autocorrelation could be explained by common climatic signals of the series [56].

The residual autocorrelation in multilevel models can be accounted for with standard corStruct classes in nlme, see p. 234 in [42]. Here, we account for the three annual-lag autocorrelation updating multilevel model in trwf with an auto-regressive structure for time lags ≥ 2, see Appendix A.3. This is developed with correlation argument in the update function. Adequacy of the fitted correlation model is tested with diagnostic plots of ACFs applied to normalized fluctuations. Choleski decomposition of lme is implemented to normalize the fluctuations, see Appendix A.2. This is controlled with resType=‘n’ argument in ACF. We also test improvements in parameters of the correlation model using the in-package anova method.

> trwfAC <- update(trwf,

           correlation = corARMA(p = 1, q = 1))

> plot(ACF(trwfAC, maxlag = 10,

     resType = ‘n’),

    alpha = 0.01) ## Fig (3), middle panel

> anova(trwf, trwfAC)

       Model df       AIC       BIC    logLik    Test  L.Ratio p-value

trwf       1 13 1425.193 1488.797 -699.5963

trwfAC    2 14 1292.074 1360.571 -632.0371 1 vs 2 135.1184  <.0001

The ACF plot indicates fluctuations in trwfAC to be uncorrelated (Fig 3, middle panel), and the anova output evinces that accounting for the residual autocorrelation improves parameters of the multilevel model (p ≤10^−4^).

## 7 Aridity-index fluctuations

The Annual Aridity Index (AAI) is a good indicator of seasonal drought. This is constructed under the assumption that 1°C of monthly temperature amounts to 2mm month^−1^ of evaporation [57, 58] helping to identify periods of relative water surplus or deficit. modelFrame can model and detrend series of AAI processing the meteorological MDDS. Formulating parameters to model aridity indexes from Prec and Temp data sets using modelFrame is similar to what was explained for modeling tree-growth fluctuations. Here, new functions moveYr and wlai are specified in fn argument (Table 2). The moveYr function transforms time units into climatic variables for the years considered begin with October (default ini.mnt = ‘Oct’, which is the beginning of meteorological year in Spain). The wlai function calculates square-root annual aridity indexes (default sqt = TRUE). Both functions are evaluated at the year level with the lv argument. Fluctuations in the aridity-index are extracted using alternative lmeForm formula. This is a linear expression developed to detrend square-root aridity indexes, see Appendix A.1.3. Default Restricted Maximum Likelihood (REML) method is maintained.

> aif <- modelFrame(list(Prec, Temp),

            fn = list(’moveYr’,’wlai’),

            lv = list(’year’,’year’),

          form = ‘lmeForm’)

> summary(aif)

Linear mixed-effects model fit by REML

 Data: fixed

      AIC        BIC    logLik

44.47932 57.40416 -17.23966

#…

Fixed effects: AI ˜ year

                     Value Std.Error DF   t-value p-value

(Intercept) -18.928112  3.648494 97 -5.187925        0

year            0.009985  0.001842 97  5.420766        0

#…

**Fig 4:**
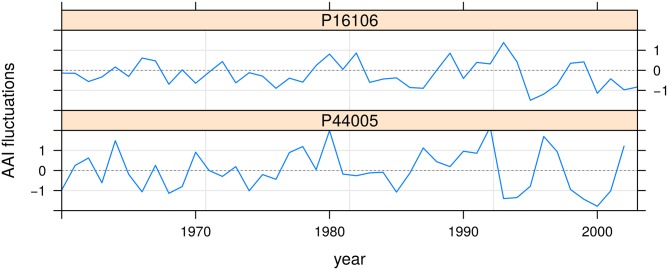
Normalized aridity-index fluctuations in aif object. Labels indicate level-codes in the plot factor.

> plot(aif) ## Fig (4)

In this case, the fixed effects terms of the aridity-index model are significant (p ≈ 0) suggesting that drought increased throughout the study area during the 50 years prior to sampling, even though the slope is very low (9.9 × 10^−3^). Detrended plot-level aridity indexes fluctuate around zero over time and display constant variance (Fig 4).

Similarly to the TRW modeling, we compare parameters of the multilevel model in aif with parameters of an lm model without grouping structure. We also inspect presence of serial patterns in the aridity-index fluctuations.

> aifl <- lm(AI ˜ year,

      data = getData(aif))

> anova(aif, aifl)

     Model df       AIC      BIC     logLik    Test   L.Ratio p-value

aif      1  5 44.47932 57.40416 -17.23966

aifl     2  3 42.43269 50.18759 -18.21635 1 vs 2 1.953366   0.3766

> plot(ACF(aif, maxlag = 10, resType = ‘n’),

    alpha = 0.01) ## Fig (3), lower panel

Parameters in the multilevel model in aif exhibit non-significant differences with regard to parameters in the lm model (p ≈ 0.38) suggesting that more extensive meteorological records are needed to properly account for plot-level variability within the multilevel model. On the other hand, the ACF plot of normalized fluctuations in aif indicates no significant autocorrelations (Fig 3, lower panel). Considering that the fixed-effects parameters in aif are significant and that the corresponding fluctuations are not autocorrelated, we use the aif model during the final process of multivariate comparison.

### 7.1 Multivariate comparison

The BIOdry package can measure relationships and patterns between classes of the dendroclimatic fluctuations by computing Mantel correlograms (Appendix A.5). This comparison is performed by evaluating the muleMan function. Here, we use this function to correlate dissimilarity matrices between fluctuations in the trwf and the aif objects which have common levels in the plot factor. We test significances of the multivariate correlations by repeatedly permuting the standardized dissimilarity matrices by rows and columns with the nperm argument.

> mcomp <- muleMan(trwf,

    cd = aif,

   nperm = 10^3^)

**Fig 5:**
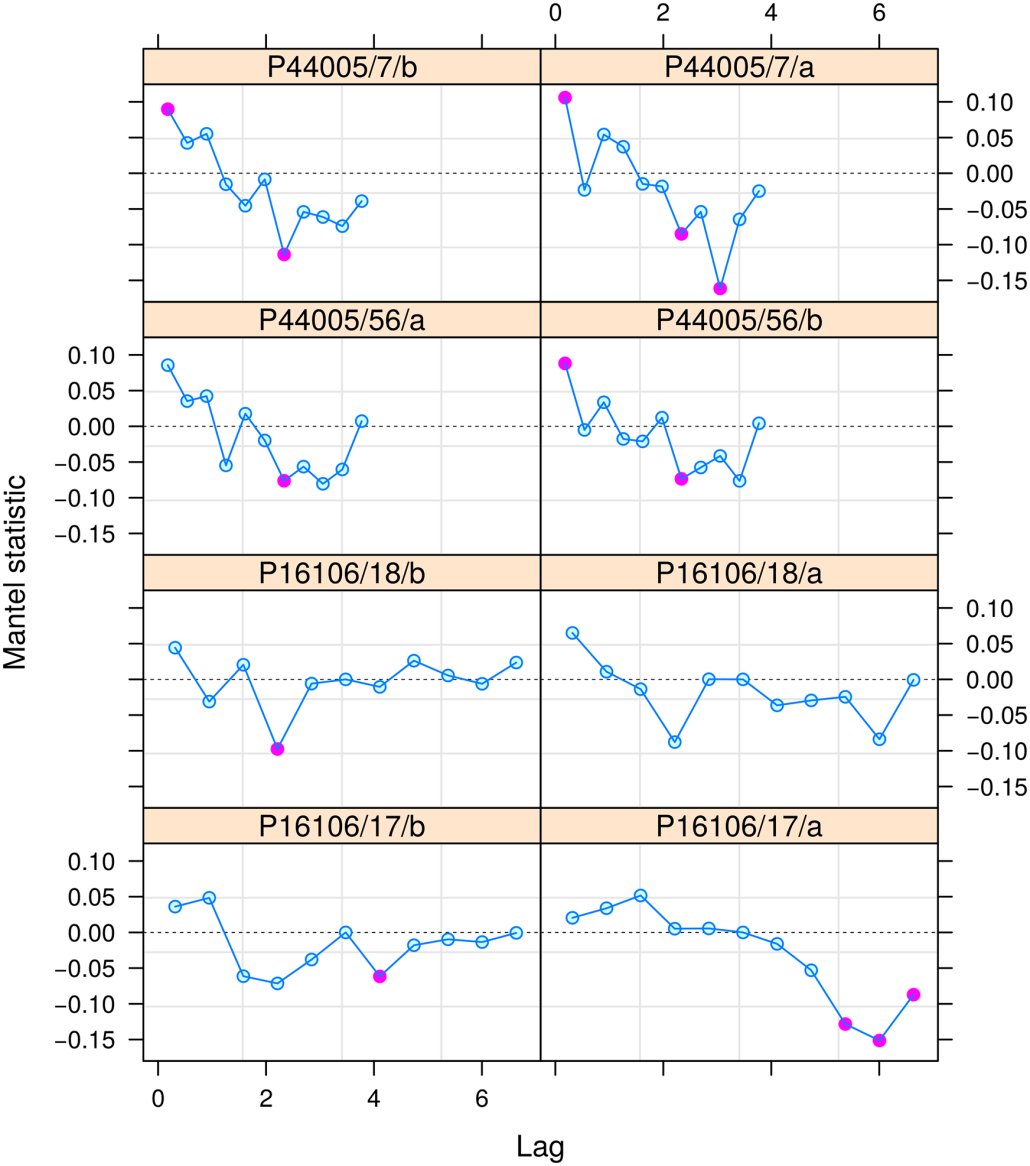
Multivariate correlations between the TRW chronology and the AAI fluctuations. Red circles indicate significant correlations (p ≥ 0.05).

> plot(mcomp) ## Fig (5)

The plot-level panels suggest that relationships between fluctuations in trwf and aif depend on the within-plot hierarchy, and plot location is the most important driver of dendroclimatic correlations (Fig 5). Within-plot variability affects the patterns of the computed correlations over time, while within-tree variability influences correlation trends and scales across the computed distance classes. Trends in the two sites exhibit oscillating pulses, and pulse frequency was significant for distance classes around 2 or 6. Although dendroclimatic interactions are stronger in East-Central Spain (P16106), they are more regular in Northern Spain (P44005) than in East-Central Spain (P16106). The Mantel correlogram also illustrates that dendroclimatic relationships are affected by within-core variability, such as radial-increment morphologies in this case. The core replicates of the same trees indicates both significant and no significant responses to drought (Fig 5, filled and empty circles, respectively), and discrepancies are more evident in P16106 than in P44005.

## 8 Conclusion

The BIOdry package is an useful tool for multilevel dendroclimatic modeling by integrating recursive evaluation of one-level functions, multilevel modeling, and multivariate comparison of dendroclimatic fluctuations. A larger community of researchers will find it relatively easy to use, as it only requires implementation of the modelFrame and muleMan functions. Most arguments in other functions of the same package, or in the nlme or the ecodist libraries, can be implemented with the same modelFrame and the muleMan functions. The implementation of the functionalities within the BIOdry package to study the effects of dendroclimatic factors on forest-ecosystem dynamics could be greatly relevant for managing natural forests or monocultures, or for understanding the role of species mixtures in forestry. In the latter case, effects from dendroclimatic factors representing complementary resource uses (root stratification, nutrients, facilitative improvements, etc.) or even commercial productivity might be modeled with the package. We hope that other scientists can implement the BIOdry package to study the effect of such factors on many species and at different successional stages.

## A Appendix: Multilevel and multivariate analyses with the BIOdry package

### A.1 Trend subtraction

Currently, the R-package BIOdry has two detrending formulas:‘tdForm’ and ‘lmeForm’. The first formula uses growth theory to subtract trends in organic variables. The second formula is a more flexible linear expression developed to detrend normalized aridity indexes and to formulate further linear relationships in MDDS: e.g. gropedData structures for panel plotting.

#### A.1.1 Growth theory

Ontogenetic component of organic growth (e.g. tree growth) behaves like a mechanistic open system with inputs and outputs of energy [40, 59, 60]. Diverse growth equations have been postulated on this basis to model, for instance, forest growth [39, 61–63], with most of the proposed equations being particular cases of following log-linear expression [41]:

$$\begin{array}{c} \text{ln}\left(Y^{\prime }\right)=\beta_{0}+\beta_{1}\;\text{ln}\left(Y_{t}\right)-\beta_{2}\;f\left(t\right) \end{array}$$ (1)

where ln is logarithm natural; $Y^{\prime }$ is the relative growth of the organism; *β* are parameters to be fitted; **Y** is cumulative organic growth; and **t** is the time. *f*(**t**) is a function of time which can be ln(**t**) or **t**. When *f*(**t**) = **t**, Eq (1) is called a Time-Decline Form (TD), and when *f*(*t*) = ln(*t*) then the equation is a Logarithmic Time-Decline Form (LTD). Even though growth theory has provided theoretical balance equations, such as the TD or the LTD forms, to detrend tree growth series, conventional dendroclimatical software implements particular cases of growth or arbitrary equations, such as polynomials or even straight lines [2, 64]. These procedure has been successful in subtracting fluctuations and signals from dendrochronologic analysis [54]. Growth equations can help to enhance detrending process in dendrochronolgical research.

#### A.1.2 Multilevel detrending of MDDS

The ‘tdForm’ method in BIOdry package is a linear generalization of Eq (1) which considers either time units or classification factors in MDDS as random effects. This method function has the following structure:

$$\begin{array}{cc} \text{ln}({\text{Y'}}_{\;\cdots \;ic,\;t}) & =A_{\;\cdots \;ic}+B_{\;\cdots \;ic}\;\text{ln}\left({\text{Y}}_{\;\cdots \;ic,\;t}\right)-C_{\;\cdots \;ic}\;f\left(\text{t}\right)+\epsilon_{\;\cdots \;ic,\;t} \end{array}$$ (2)

$$\begin{array}{c} A_{\;\cdots \;ic}=\beta_{0}+\upsilon_{\;\cdots ,\;0}+\upsilon_{\;\cdots \;i,\;0}+\upsilon_{\;\cdots \;ic,\;0} \end{array}$$ (3)

$$\begin{array}{c} B_{\;\cdots \;ic}=\beta_{1}+\upsilon_{\;\cdots ,\;1}+\upsilon_{\;\cdots \;i,\;1}+\upsilon_{\;\cdots \;ic,\;1} \end{array}$$ (4)

$$\begin{array}{c} C_{\;\cdots \;ic}=\beta_{2}+\upsilon_{\;\cdots ,\;2}+\upsilon_{\;\cdots \;i,\;2}+\upsilon_{\;\cdots \;ic,\;2} \end{array}$$ (5)

$$\begin{array}{c} \epsilon_{\;\cdots \;ic}∼N\left(0,R\right), \end{array}$$ (6)

where the *β* are the model parameters, **υ**_⋯_ = (*υ*_⋯,0_, *υ*_⋯,1_, *υ*_⋯,2_)^*T*^ are vectors of outermost levels of grouping, **υ**_⋯*i*_ is a vector of tree random effects nested in the outermost levels of grouping, and **υ**_⋯*ic*_ is a vector of core random effects nested in trees that are nested in the outermost levels. The vector **ϵ**_⋯*ic*,*t*_ contains the within-group residual errors *ϵ*_⋯*ic*,*t*_ and follows a multivariate normal distribution **ϵ**_⋯*ic*,*t*_ ∼ *N*(**0**, **R**).

#### A.1.3 Trends in aridity indexes

Algorithms in BIOdry can construct Walter-Lieth diagrams and compute annual aridity indexes as the ratios between dry and wet areas in the diagrams. The inputs are MDDS of monthly average temperatures (°C) and monthly precipitation sums (mm). This integrates seasonal precipitations and temperatures to identify periods of relative water surplus and deficit [65]. Square root transformation of the aridity indexes are usually stationary [20]. Transformed aridity indexes also exhibit weak linear trends [52] that can be subtracted with simple linear equations, without further filtering the low and high frequencies observed in the raw precipitation and temperature records [43]. Autocorrelation in climatic proxies has been a frequent problem in dendroclimatology [47]. The square root transformed indexes use are weakly autocorrelated [52].

The ‘lmeForm’ method implements a flexible linear formula, which was developed to enable users formulate their own multilevel linear expressions. For instance, form = ‘lmeForm’ can be used to detrend the aridity indexes. In such a case, the ‘lmeForm’ method formula would have the following structure:

$$\begin{array}{c} \sqrt{{\text{AAI}}_{\;k,\;t}}=D_{k}+E_{k}\;\text{t}+\omega_{\;k,\;t} \end{array}$$ (7)

$$\begin{array}{c} D_{k}=\gamma_{0}+\tau_{\;k,\;0} \end{array}$$ (8)

$$\begin{array}{c} E_{k}=\gamma_{1}+\tau_{\;k,\;1} \end{array}$$ (9)

$$\begin{array}{c} \omega_{\;k,\;t}∼N\left(0,R\right)\text{,} \end{array}$$ (10)

where the *γ* are the model parameters, ***τ***_*k*_ = (*τ*_*k*,0_, *τ*_*k*,1_)^*T*^ is the vector of location random effect. The vector ***ω***_*k*,*t*_ contains the location-level residual errors *ω*_*k*,*t*_ and follows a multivariate normal distribution ***ω***_*k*,*t*_ ∼ *N*(**0**,**R**). These are ordered on time and depict climatic fluctuations.

#### A.1.4 Back-transformation of model parameters

In some applications involving ecological analysis of dendroclimatic variables, it might be necessary to back-transform tree-growth and aridity-index predictions from Eqs (2) and (7) to the original scales. It is well known that exponential and power transformations could introduce biases and correction factors must be applied [66, 67]. However, it is not an objective of the BIOdry package to find how correction factors could best be incorporated in mixed effects models. Analyses with the package focus on comparing dendroclimatic fluctuations without the need of back-transforming variables and the mixed-effects models include no correction factors. Therefore, inferences with the package apply only to the untransformed values of the parameters.

### A.2 Residual normalization

Multilevel fluctuations in modelFrame objects are normalized via Choleski decomposition of the residual variance-covariance matrix, see p. 239 in [42]. For the innermost level of Residuals of Annual Biomass Increment (RABI) the normalized residuals are defined as follows:

$$\begin{array}{c} \epsilon_{\;\text{norm},\;c,\;t}={\left({C_{\;c,\;t}}^{T}\right)}^{-1}\left(y_{\;c,\;t}-X_{\;c,\;t}\hat{\beta }\right)\text{,} \end{array}$$ (11)

where ${C_{i}}^{T}$ is the lower triangle of the Cholesky decomposition of the variance-covariance matrix **V**_*i*_, **X**_*i*_ is the design matrix, $\hat{\beta }$ contains the parameter estimates and **y**_*i*_ is the vector of observed responses.

### A.3 Autocorrelation function

Autocorrelation of normalized fluctuations in multilevel models is assessed with ACF, see p. 183 in [28]. As an example, the autocorrelation model for the lowest level of ***ϵ***_⋯*ic*,*t*_ normalized residuals is

$$\begin{array}{c} \hat{\rho }\left(l\right)=\frac{\sum_{c=1}^{n_{i}}\sum_{t=1}^{n_{c}-l}\epsilon_{\;c,\;t}\epsilon_{\;c,\;(t-l)}/N\left(l\right)}{\sum_{c=1}^{n_{i}}\sum_{t=1}^{n_{c}}\epsilon_{\;c,\;t}^{2}/N\left(0\right)} \end{array}$$ (12)

where *l* is the lag in years, and *N*(*l*) is the number of residual pairs defining the numerator of $\hat{\rho }\left(l\right)$. Any correlation structure of nlme package can be implemented in modelFrame function to model residual autocorrelation. For the case of dendroclimatic data, autocorrelation in MDDS is modeled with an autoregressive-moving average models ARMA (1,1), which have exponentially decaying auto-correlation functions for lags ≥ 2. For the lowest level of the ***ϵ***_⋯*ic*,*t*_ normalized residuals the autocorrelation model is

$$\begin{array}{c} \epsilon_{c,\;t}=\phi \epsilon_{c,\;(t-1)}+\theta a_{c,\;(t-1)}+a_{c,\;t}\text{,} \end{array}$$ (13)

where *ϕ* was the autoregressive parameter; *θ* was the moving average parameter; and *a*_*c*,*t*_ was the noise term, see p. 128 in [50].

### A.4 Variance function

Heteroscedasticity and serial autocorrelation of $\hat{\epsilon_{\;\cdots \;ic,\;t}}$ and Residuals of Annual Aridity Index (RAAI) can be modeled with correlation and weights arguments [42] in modelFrame function. However, such modeling would take a long time depending on the complexity of the classification factors in the MDDS. For example, the variance model for the innermost level of ***ϵ***_⋯*ic*,*t*_ normalized residuals is:

$$\begin{array}{c} \text{Var}\left(\epsilon_{\;c,\;t}\right)=\sigma^{2}{\left(\varrho_{1}+{|\nu_{\;c,\;t}|}^{\varrho_{2}}\right)}^{2}\text{,} \end{array}$$ (14)

where *σ*^2^ was the variance; $\varrho_{1}$ and $\varrho_{2}$ were constants; *ν*_*c*,*t*_ had the same structure of the fixed effects from Eqs (2) and (7).

### A.5 Multivariate comparison

Mantel correlograms between two MDDS with a common classification factor are established by comparing distances in one of the MDDS with sets of binary model matrices specifying membership in particular classes of the other MDDS. Euclidean distances from both MDDS are standardized to z-scores by subtracting the means from individual distances and then dividing the differences by the corresponding standard deviations. The Mantel statistic is

$$\begin{array}{c} r\left(d\right)=\frac{\sum_{i}^{n}\sum_{j}^{n}w_{ij}z_{ij}}{\sum_{i}^{n}\sum_{j}^{n}w_{ij}} \end{array}$$ (15)

where *d* is the distance class from one of the MDDS; *z*_*ij*_ is the distance between each pair *i* and *j* from the other MDDS; *w*_*ij*_ is a weight for the pair: typically 1 if *z*_*ij*_ is in *d* and 0 if it is not. The number of classes *d* is calculated with the Sturges rule [29]:

$$\begin{array}{c} d=1+3.3\cdot {\text{log}}_{10}(m) \end{array}$$ (16)

where *m* is the number of distances in the upper-triangular binary model matrix.
